# A Comparison of DNA-Methylation during Protoplast Culture of Ponkan Mandarin (*Citrus reticulata* Blanco) and Tobacco (*Nicotiana tabacum* L.)

**DOI:** 10.3390/plants13202878

**Published:** 2024-10-15

**Authors:** Lun Wang, Jiaojiao Zhang, Xiaoyong Xu

**Affiliations:** 1 College of Horticulture and Landscape Architecture, Yangzhou University, Yangzhou 225009, China; jiaojiaozhang2021@126.com; 2 Key Laboratory of Plant Functional Genomics of the Ministry of Education, Agricultural College of Yangzhou University, Yangzhou 225009, China

**Keywords:** regeneration, MSAP, hypermethylation, gene expression

## Abstract

The epigenetic variation in protoplast regeneration is a topic that has attracted interest recently. To elucidate the role of DNA methylation in the regeneration of protoplasts from the ponkan (*Citrus reticulata*), this study employs the methylation-sensitive amplification polymorphism (MSAP) molecular marker technique to analyze changes in DNA methylation levels and patterns during the isolation and culture of protoplasts from ponkan and tobacco. Additionally, differential DNA methylation fragments are cloned, sequenced, and subjected to bioinformatics analysis. The results reveal that, for non-regenerable ponkan mesophyll protoplasts, DNA methylation levels increase by 3.98% after isolation and then show a trend of initial decrease followed by an increase during culture. In contrast, for regenerable ponkan callus protoplasts and tobacco mesophyll protoplasts, DNA methylation levels decrease by 1.75% and 2.33%, respectively, after isolation. During culture, the DNA methylation levels of ponkan callus protoplasts first increase and then decrease, while those of tobacco mesophyll protoplasts show an opposite trend of initial decrease followed by an increase. Regarding DNA methylation patterns, ponkan mesophyll protoplasts exhibit primarily hypermethylation changes accompanied by a small amount of gene demethylation, whereas ponkan callus protoplasts are dominated by demethylation changes with some genes undergoing hypermethylation. The methylation exhibits dynamic changes in protoplast isolation regeneration. By recovering, cloning, sequencing, and performing BLASTn alignment analysis on specific methylation modification sites in the ponkan, 18 DNA sequences with high homology are identified which are found to be involved in various biological functions, thereby establishing a foundational basis for genetic editing in protoplasts.

## 1. Introduction

Protoplasts, defined as cells lacking cell walls, exhibit high versatility in research applications. They have a wide range of applications in genetic manipulation and cellular studies, including somatic hybridization [1], CRISPR/Cas9 genome editing [2,3] or genetic transformation [3,4,5,6], transient gene expression [5,7,8,9], protein interaction [10,11,12,13], signal transduction [14], and synthetic biology [15,16,17]. A fundamental prerequisite for the application of this technology is the ability of the isolated protoplasts to divide and regenerate [15].

Leaves are commonly used as explants for protoplast isolation. However, the limited regeneration capacity of many plant protoplasts, particularly those derived from leaves (mesophyll), poses a significant challenge to the full harnessing of the potential of protoplast technology for crop improvement and other applications. Numerous plant mesophyll protoplasts currently exhibit either an inability or difficulty to regenerate into whole plants [18]. Consequently, the regeneration ability of protoplasts has become a focal point of research [19,20,21,22,23].

Previous studies have established that oxidative stress serves as a physiological barrier to the regeneration of plant protoplasts [24,25], while the genes related to cell proliferation, cell wall synthesis, and oxidative stress may contribute to the barrier to the regeneration of plant protoplasts. The genetic factors contributing to this are complex, for example, the DNA transcription level involves the DNA variation including somaclonal variation and germline variation. In addition, the relationship between methylation and protoplast regeneration has also been explored. DNA methylation varies in a highly gene- and chromosome-differential manner during in vitro differentiation and regeneration [26,27]. Nuclear DNA can be modified by post-replicative methylation of its cytosine residues, with over 80% of cytosines in CpG sites being methylated in most plants [28]. The base 5-hmC did not primarily arise as a product of oxidative DNA damage following protoplast culture [29]. Pavla et al. (2012) [29] compared the DNA methylation changes of cucumber protoplasts (callus proliferation) and kale protoplasts (regenerated plants) during 72 h of culture, and found that the cucumber DNA methylation level of freshly isolated protoplasts was not different from that of kale. However, during the culture process, the DNA methylation level showed a decreasing trend, and the DNA methylation content of the cucumber was significantly lower than that of kale. The study of the methylation variation of the protoplast of *Ulva reticulata* has revealed the distinct expression of cytosine methylation and is thus correlated with the differential morphogenesis of plants regenerated from cultured cells [30].

The precise location of methylation variation within the genome and the underlying molecular mechanisms responsible for variability in protoplast regeneration remain to be investigated, as they are crucial for epigenetic editing in protoplasts [31,32,33,34]. With the advancement of epigenetic research and the proliferation of associated technologies, investigating molecular changes during protoplast regeneration from an epigenetic perspective is anticipated to unravel these underlying mechanisms [35]. Significant advancements have been made in the biotechnology of horticultural crops, particularly in somatic hybridization and genetic transformation studies involving protoplasts [36,37,38,39]. Citrus protoplasts isolated from callus tissue exhibit the ability to continue dividing and regenerating [40]. Mesophyll protoplasts, on the other hand, are unable to divide and regenerate under current culture conditions and are thus unsuitable for citrus genetic improvement. Consequently, citrus protoplasts provide an ideal model for investigating differences in protoplast regeneration capacity. Furthermore, the culture of tobacco mesophyll protoplasts serves as another model system for studying protoplast regeneration.

Given the aforementioned challenges, the present study aims to utilize the methylation-sensitive amplification polymorphism (MSAP) molecular markers to investigate the levels and patterns of methylation/demethylation during the isolation and culture of citrus mesophyll protoplasts, callus protoplasts from the same variety, and tobacco mesophyll protoplasts. Differential DNA methylation fragments are cloned, sequenced, and analyzed using bioinformatics tools in an effort to explore the molecular mechanisms underlying the differences in citrus protoplast regeneration.

## 2. Results

### 2.1. DNA Methylation Changes during Protoplast Isolation and Culture Process

#### 2.1.1. Observations during Protoplast Culture and Selection of MSAP Analysis Nodes

**Figure 1:**
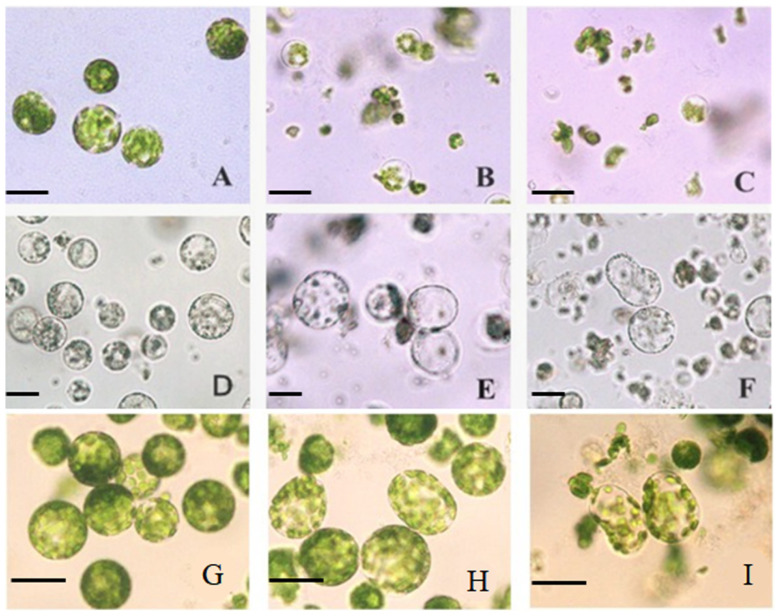
Observation of freshly isolated and cultured protoplasts from citrus and tobacco under bright field. (**A**–**C**) Citrus mesophyll protoplasts after 0, 3 and 6 days of culture; (**D**–**F**) citrus callus protoplasts after 0, 4, 8 days of culture; (**G**–**I**) tobacco mesophyll protoplasts after 0, 1, 2 days of culture, The scale bars = 20 μm.

Growth changes during the protoplast culture of ponkan and tobacco to determine the nodes for MSAP analysis were observed. Both newly isolated mesophyll and callus protoplasts had intact cell membrane structures and high vitality (Figure 1A,D,G). However, during subsequent cultures, there were significant differences in the growth and development of protoplasts with different regeneration abilities. For non-regenerable citrus leaf protoplasts, over 75% of them began to rupture and die on the third day of culture (Figure 1B,H), and almost all of them ruptured and died on the sixth day (Figure 1C). Hence, leaf protoplasts are not suitable for MASP marker analysis. During the culture process, only a small number of callus protoplasts ruptured and died. Additionally, on the fourth day of culture, individual protoplasts exhibited an elliptical morphology (Figure 1E). On the eighth day, over 95% of the protoplasts became elliptical, while some individual protoplasts began to divide (Figure 1F), indicating that this node is the time point at which the callus tissue protoplasts initiate cell division. Based on this, the first and second day of tobacco mesophyll protoplast culture are the time points for cell wall regeneration and cell division initiation, respectively.

#### 2.1.2. Analysis of Genomic DNA Methylation Levels during Protoplast Isolation and Culture Process

In the ponkan, 49 primer combinations were used to amplify a total of 1257 bands in each sample. Among these, 1004 bands were amplified in the leaf and mesophyll protoplasts of the ponkan, and 972 bands were amplified in the embryonic callus and protoplasts of the ponkan. For the tobacco, a total of 558 bands were generated by amplification with 18 pairs of primers (Table 1). The DNA methylation level of non-renewable ponkan leaf protoplasts increased by 3.98% after isolation, and, during the culture process, it first decreased and then increased. The DNA methylation levels of ponkan callus protoplasts and tobacco mesophyll protoplasts decreased by 1.75% and 2.33%, respectively, after separation. During the culture process, the protoplasts of ponkan callus tissue first increased and then decreased in DNA methylation levels, while the protoplasts of tobacco mesophyll tissue first decreased and then increased. The results show that the DNA methylation level of citrus leaves was above 26.2%.

#### 2.1.3. Analysis of DNA Methylation Patterns during Protoplast Isolation and Culture Process

**Figure 2:**
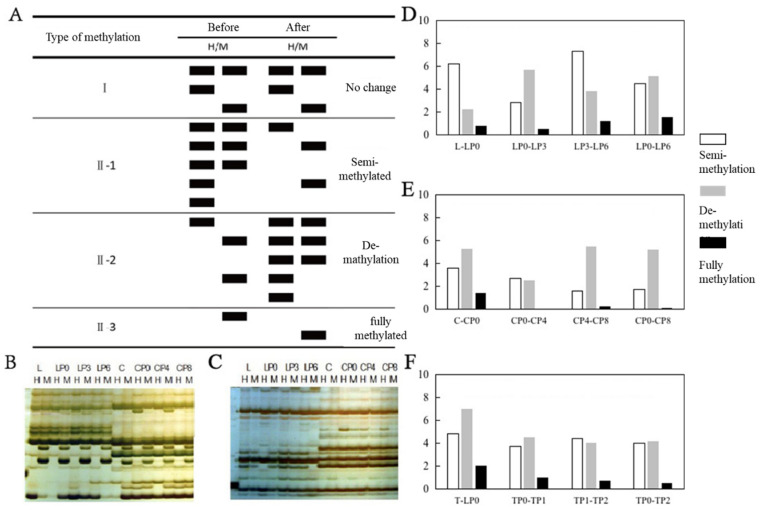
Changes in DNA methylation patterns during protoplast isolation and culture. (**A**) Types of methylation pattern changes during protoplast isolation and culture, (**B**) MSAP profile of the primer EA11/HM + TAC, (**C**) primer EA05/HM + TAT MSAP atlas, (**D**) changes in DNA methylation patterns during the isolation and culture of ponkan mesophyll protoplasts, (**E**) changes in DNA methylation patterns during the isolation and culture of ponkan callus protoplasts, and (**F**) changes in DNA methylation patterns of tobacco protoplast isolation and culture.

By comparing the differences in MSAP bands between two time points, it was found that changes in DNA methylation patterns can be mainly classified into two categories: homozygosity and polymorphism. Type I in Figure 2A represents a singlet pattern, indicating that the methylation status of the CCGG site remains unchanged at certain stages during the separation and culture process of the protoplast. The polymorphic loci indicate that the MSAP band patterns changed during these two periods and can be divided into three subtypes: II-1 represents a pattern of methylation, where the preceding period is non-methylated and the successive period is either fully methylated or semi-methylated, II-2 represents a pattern of demethylation, where the preceding period is either fully methylated or semi-methylated and the subsequent period is semi-methylated or non-methylated, and II-3 represents a transition into full methylation, where the inner cytosine of the CCGG site undergoes full methylation in the previous period and becomes fully methylated in the outer cytosine in the successive period, with relatively few changes in this pattern. Figure 2B,C shows the MSAP profiles of partial primer combinations.

Based on the MSAP map obtained from 49 pairs of primers for selective amplification, statistical analysis was conducted on the changes in DNA methylation patterns during the isolation and culture of ponkan protoplasts at different stages. As shown in Figure 2D,E, DNA methylation and demethylation events occurred at different stages, but there were significant differences between different materials. Before and after the separation of protoplasts from the leaf flesh of the ponkan, the proportion of methylation was higher than that of demethylation, while the proportion of protoplasts from callus tissue was the opposite. On the third day of mesophyll protoplast culture, compared with newly separated protoplasts, mainly demethylation occurred. On the fourth day of culture, there was a small difference in methylation levels between callus protoplasts and newly separated protoplasts, with 2.7% of the sites undergoing methylation and 2.39% undergoing demethylation. On the sixth day of culture, compared to the third day, 7.17% of the sites in the mesophyll protoplasts underwent methylation, 3.58% underwent demethylation, and the callus protoplasts mainly underwent demethylation. On the sixth day of mesophyll protoplast culture, there were more demethylation sites compared to when it was first isolated, while, in callus protoplasts, most of the methylation sites were demethylated.

For tobacco mesophyll protoplasts, as shown in Figure 2F, the changes in DNA methylation patterns before and after protoplast isolation were consistent with the changes in protoplasts from citrus callus tissues. However, the changes in DNA methylation patterns during its culture process were similar to those in ponkan leaf protoplasts. From this, it can be seen that the acquisition of pluripotency of protoplasts with regenerative abilities after separation is related to a decrease in DNA methylation levels. However, differences in DNA methylation levels and patterns during the culture process may be related to species and protoplast culture time.

### 2.2. Specific Methylation Modification Site Sequence Analysis

Specific methylation modification sites in the ponkan citrus genome were recovered, cloned, sequenced, and, ultimately, 30 sequences were successfully recovered. BLASTn alignment analysis was conducted on the Sweet Orange Database website (http://citrus.hzau.edu.cn/), and 18 out of 30 sequences showed high homology with the sweet orange genome alignment (Table 2). According to the functional annotation of matching homologous sequences, it was found that these specific methylation sequences include protein coding sequences (P8, P14, P20, biosynthetic pathways) and protein kinases (P9, P19, P25, P28, regulatory pathway). There are various types of DNA sequences, including hydrolytic proteases (P15, P55, regulatory pathway), ubiquitin proteases (P23, regulatory pathway), RNA polymerase (P30, regulatory pathway), endonucleases (P46, biosynthetic pathway), disease-resistant proteins (P53, biosynthetic pathways), and repeat sequences.

## 3. Discussion

Previous studies have shown that MSAP technology is suitable for analyzing methylation sites at the whole plant genome level and has been widely applied to the study of various aspects of plant epigenetics [41,42,43]. However, it is important to note that the homolytic enzyme used in MSAP technology is limited in its potential to recognize cytosine methylation at the “CCGG/GGCC” site. Consequently, it is unable to detect simultaneous methylation of both double-stranded outer cytosine and double-stranded inner and outer cytosine. Evidence of the aforementioned methylation types recognized by the two enzymes is limited [42].

In this study, we used MSAP technology to investigate the methylation level of the CCGG sites during the isolation and culture of ponkan protoplasts. The results showed that the DNA methylation level of citrus leaves was about 26.2%, which was close to the DNA methylation level of the Shatian pomelo (21.6–26.0%) [44].

The DNA methylation level in the callus tissue of the ponkan is higher than that in the leaves, a phenomenon which may be related to the degree of differentiation among plant tissues [35,45]. Furthermore, the methylation level of newly isolated ponkan mesophyll protoplasts is higher than that found in the leaves, indicating that some genes in the leaves regulating plant regeneration undergo methylation during the process of cell wall removal and the protoplast forming. This may be involved in the enhancement of transposon inactivation, leading to gene silencing [27]. Although both callus tissue and its protoplasts are capable of regenerating into plants, the methylation level decreases during the separation and culture of protoplasts, reaching its lowest point during the first entry into the division phase. A comparison of rice callus tissue and its regenerated plants has also revealed a reduction in methylation levels, occurring during the tissue culture stage [46]. The separation of protoplasts represents a stress process, and previous studies have shown that the level of reactive oxygen species (ROS) affects protoplast regeneration [25]. The genome variation may also have an influence on the methylation pattern [20]. To eliminate the possibility of tissue specificity in the materials, we conducted the MSAP analysis on tobacco leaves and their protoplasts, both of which are capable of regeneration. We demonstrate that the methylation level also decreased after protoplast separation, consistently with the results observed in ponkan callus protoplasts.

DNA methylation patterns seem to be related to the acquisition and maintenance of totipotency in plant protoplasts, involving the activation of silenced genes [47]. By comparing MSAP banding patterns between stages, changes in DNA methylation patterns can be classified into two types: monomorphic methylation sites and polymorphic methylation sites. Based on the occurrence of hypermethylation and hypomethylation, as well as potential shifts in methylation types between two consecutive stages, polymorphic sites can be further subdivided into different subclasses. These results indicate the nuanced dynamics of the methylation changes during protoplast formation and underline the drastic changes occurring during protoplast isolation [48].

In mesophyll protoplasts, hypermethylation is the dominant change, accompanied by a small amount of hypomethylation of certain genes. In contrast, callus protoplasts exhibit, predominantly, hypomethylation, with some genes undergoing hypermethylation. MSAP analysis of *Arabidopsis thaliana* leaves and their protoplasts has revealed that 13% of methylation sites show alterations in methylation patterns between leaves and protoplasts [23,49]. BLAST analysis indicated that these sequences are located at sites on various chromosomes, as well as in regulatory and coding regions, chloroplast DNA, and transposable elements.

As methylation serves as a regulatory mechanism for controlling gene expression during plant development and cell differentiation, although the specific processes of how methylation modulates gene expression are not fully understood [49], sequence analysis was conducted on a subset of methylation polymorphic fragments. Through BLASTn alignment in citrus genome databases, we identified homologous sequences for 18 out of 30 ponkan (*Citrus reticulata*) sequences. Based on the functional annotations of the matched homologous sequences, we found that these specific methylation sequences encompass a diverse array of DNA sequences, including those encoding proteins, protein kinases, hydrolytic proteases, ubiquitin proteases, RNA polymerases, glycosyltransferases, nuclear matrix proteins, endonucleases, disease resistance proteins, and repetitive sequences. The varying degrees of methylation modifications observed in these genes during protoplast isolation and regeneration from different materials suggest their potential involvement in plant regeneration.

Approximately 60–90% of CpG sites undergo methylation, with unmethylated CpGs predominantly located in gene promoter regions during ponkan callus protoplast isolation. In contrast, high levels of DNA methylation in *Arabidopsis thaliana* mainly occurs in repetitive element sequences, such as centromeric regions, where approximately one third of the genes in transcribed regions exhibit high DNA methylation, while only around 5% of gene promoters are methylated [49,50]. Methylation in promoter regions can lead to transcriptional silencing at the initiation stage, while methylation modifications in gene coding regions have more complex biological functions. DNA methylation in transcribed regions can prevent transcription initiation, and CHG methylation can hinder transcription elongation. Methylation in gene coding regions may be an intrinsic byproduct of transcription that may need to be restricted to CG sites to avoid adversely affecting transcription [51,52]. It has been found that a considerable proportion of DNA methylation exists in both coding genes and transposon sequences in the *Arabidopsis* genome, and the degree of methylation is closely related to the transcriptional activity of these sequences; therefore, it can affect plant immunity [53] and influence plant phenotypes. Over the past decade, studies have shown that methylation also occurs in the genomes of organelles such as mitochondria and chloroplasts [54,55,56]. In this study, tobacco methylation-specific fragments cloned and sequenced were identified as chloroplast DNA upon comparison with NCBI databases, enriching the diversity of detected methylation sites in higher plant genomes.

Further screening of methylation polymorphic genes during plant regeneration and analysis of their gene structures and functions will contribute to a broader and more systematic understanding of the molecular mechanisms underlying plant regeneration. In addition, the epigenome editing might help make up for deficiencies of genome editing (such as gene knockout) which have significant off-target effects and only enable the loss of a gene’s function [57].

## 4. Materials and Methods

### 4.1. Experimental Materials

Ponkan tissue was collected from the citrus germplasm repository at Yangzhou University. Based on the observation of the protoplast culture development, we collected leaves and callus tissues from the ponkan mandarin, along with their protoplasts, across three culture stages. Additionally, we gathered tobacco leaves and their protoplasts at three culture stages for MSAP analysis. The calluses derived from the leaves of the ponkan mandarin (*Citrus reticulata* Blanco) were cultured on solid MT medium supplemented with 0.15 M sucrose and 7 g L^−1^ agar, adjusted to a pH of 5.8. The seeds of both ponkan mandarin and tobacco were surface sterilized using a 10% sodium hypochlorite solution for 2 min, thoroughly rinsed with distilled water, and subsequently sown on Murashge-Tucker Medium (MT medium) containing 30 g L^−1^ sucrose.

### 4.2. Experimental Methods

#### 4.2.1. Isolation and Culture of Protoplasts

The isolation and purification of protoplasts were conducted following the method described by Xu Xiaoyong et al. [25] (2016). For the separation of callus protoplasts, 1–2 g of callus tissue was placed in a culture dish, and 2 mL of enzyme solution and 0.7 mol/L EME medium (MT basal medium plus malt extract) was added. The dish was then sealed with a sealing film and incubated under dark conditions at 28 °C for 18–20 h of enzymatic hydrolysis.

For the separation of mesophyll protoplasts, the leaves of test tube seedlings were cut into 1–2 mm wide strips and placed in a pre-added 2 mL of 0.6 mol/L EME medium. Subsequently, 2 mL of enzyme solution was added, and the mixture was sealed with a sealing film and incubated under dark conditions at 28 °C for enzymatic hydrolysis for 20–24 h. After completion of the enzyme hydrolysis, the mixture was first filtered through a 100-mesh steel sieve, and then the filtrate was filtered through a 325-mesh steel sieve, washed with CPW13, and centrifuged.

Finally, a CPW13/CPW26 interface gradient centrifugation was used, and the intermediate protoplasts were sucked out using straws. The protoplasts were then suspended in liquid culture medium MA, sealed with a sealing film, and incubated in a constant temperature incubator at 28 °C. The growth status of the protoplasts was regularly observed under an inverted microscope, and photos were taken to record the time of cell wall regeneration and entry into the first division of protoplasts. Corresponding time protoplasts were collected as experimental samples.

#### 4.2.2. Genomic DNA Extraction

Based on the time points when the protoplast exhibited 0%, 75%, and 95% corruption rates, samples were collected for analysis at the following time points: on the 0th, 3rd, and 6th day for ponkan mesophyll protoplasts, on the 0th, 4th, and 8th day for protoplasts derived from ponkan callus tissues, and on the 0th, 1st, and 2nd day for tobacco mesophyll protoplasts. The total genomic DNA of the aforementioned plant materials and the fresh leaves was extracted according to the improved CTAB method proposed by Cheng Yunjiang et al. [58]

#### 4.2.3. Genomic MSAP Analysis

The MSAP analysis was performed with reference to the method of Hao Yujin (2000) [59], with slight modifications. The primer sequences for connector, pre-amplification, and selective amplification are shown in Table 3.

##### Restrictive Enzyme Digestion

The genomic DNA of 18 materials was subjected to double enzyme cleavage using EcoRI enzyme combined with HpaII and MspI enzymes, respectively. The reaction mixture contained 4 μL of 10 × tangoTM, 0.5 μL of EcoRI (10 U/μL), 0.5 μL of HpaII (10 U/μL)/MspI (10 U/μL), 1 μL of genomic DNA (500 ng), and 14 μL of sterilized double distilled water. The mixture was incubated at 37 °C for 2.5 h and then at 80 °C for 20 min to terminate the reaction.

##### Joint Connection

The enzyme digestion mixture was added to the linking reaction system. The reaction mixture contained 20 μL of double enzyme digestion products, 4 μL of 10 × T4 buffer, 1 μL of EcoRI adapter (5 μM), 1 μL of H-M adapter (50 μM), 1 μL of T4 DNA ligase (2.5 U/μL), and 13 μL of sterilized double distilled water. The mixture was incubated overnight at 16 °C for ligation.

##### Pre-Amplification and Selective Amplification

The pre-amplification PCR mixture contained 2 μL of 10 × buffer, 1 μL of dNTPs (2.5 mM), 1 μL of EA00 (25 ng/μL), 1 μL of HM00 (25 ng/μL), 5 μL of connecting product, 0.5 μL of Taq enzyme (5 U/μL), and 9.5 μL of sterilized double distilled water. The pre-amplified product was diluted 1:30 and used as the template for selective amplification. The selective amplification mixture contained 2 μL of 10 × buffer, 2 μL of dNTPs (2.5 mM), 1 μL of EA00 (25 ng/μL), 1 μL of HM00 (25 ng/μL), 5 μL of pre-amplified product, 0.5 μL of Taq enzyme (5 U/μL), and 8.5 μL of sterilized double distilled water. The amplification program followed the method by Meng Haijun (2006) and was performed on a PCR machine.

##### Non-Denaturing Polyacrylamide Gel Electrophoresis

Non-denaturing polyacrylamide gel electrophoresis was performed at a constant power of 60 W for 30 min for pre-electrophoresis. The sample loading volume was 3–4 μL. After loading, electrophoresis was carried out at a constant power of 90 W for about 2 h. The electrophoresis was terminated when the distance between xylene blue and the bottom was about 1/3. The two glass plates were separated using a plastic spatula, followed by staining. After silver staining, observation was conducted and photographies were taken.

#### 4.2.4. Methylation-Specific Fragment Recovery, Cloning, and Sequencing

The gel was cut and transferred to a 1.5 mL centrifuge tube. A total of 400 μL of TE buffer was added, and the mixture was shaken slightly before being placed in a water bath at 80 °C for 1 h. The gel suspension was then transferred to a centrifuge column with a yellow gun head with the tip cut off and centrifuged at 12,000 rpm for 10 min. A 1/10 volume of 3 M NaAC (pH 5.2) and 2.5 times the volume of anhydrous ethanol were added to the filtrate, mixed well, and allowed to stand at −20 °C for at least 1 h. The mixture was centrifuged at 4 °C and 12,000 rpm for 10 min, and the supernatant was discarded. The mixture was washed twice with 70% alcohol, dried, and then dissolved in 10–20 μL of sterilized double distilled water.

Using the recycled fragments as templates, amplification was performed according to the primer combination and program of the original MSAP selective amplification. The PCR product was subjected to 1% agarose gel electrophoresis. If the band was clear and the molecular weight met the requirements, it was recovered and purified using the TaKaRa MiniBEST Agarose Gel DNA Extraction Kit Ver.3.0.

The connection reaction of PCR purified products was carried out according to the instructions of TaKaRa’s pMD 18-T Vector. A total of 0.5 μL of pMD18-T vector and 4.5 μL of PCR purified product was mixed well and incubated at 4 °C for 2–3 h for the connection reaction. After electrophoresis detection, the product was sent to Shanghai Shenggong Biotechnology Services Co., Ltd. for sequencing. The obtained sequence results were subjected to homology search using the BLAST (Basic Local Alignment Search Tool) method on the citrus genome website.

## 5. Conclusions

The study reveals distinct differences in DNA methylation dynamics between non-regenerable and regenerable protoplasts and suggests a potential correlation between methylation changes and the regenerative capacity of protoplasts. During culture of ponkan mesophyll protoplasts (non-regenerable), DNA methylation levels increased by 3.98%, while, in the culture of the ponkan callus protoplasts (regenerable), DNA methylation levels decreased by 1.75%. In general, ponkan mesophyll protoplasts primarily exhibited hypermethylation changes, with some genes undergoing demethylation, while the ponkan callus protoplasts were dominated by demethylation changes, with some genes undergoing hypermethylation. In addition, genomic fragments with differential methylation were explored for the gene associated with the regeneration. Three types of fragments (pertaining to biosynthetic genes, regulatory genes, or repetitive sequences) were analyzed in the context of organogenesis, embryogenesis, and oxidative stress. This research lays the foundation for genetic editing in citrus protoplasts.

## Figures and Tables

**Table 1:** DNA methylation changes in citrus/tobacco protoplasts during isolation and in vitro culture.

Samples	Total Fragments	Non-Methylation CCGG Sites	Methylation CCGG Site		
			Half Methylation Site	Full Methylation Site	Total
L	1004	741	68 (6.77%)	195 (19.42%)	263 (26.20%)
LP0	1004	701	71 (7.07%)	232 (23.11%)	303 (30.18%)
LP3	1004	732	63 (6.27%)	209 (20.82%)	272 (27.09%)
LP6	1004	696	72 (7.17%)	236 (23.51%)	308 (30.68%)
C	972	688	55 (5.66%)	229 (23.56%)	284 (29.22%)
CP0	972	705	57 (5.86%)	210 (21.60%)	267 (27.47%)
CP4	972	702	61 (6.28%)	209 (21.50%)	270 (27.78%)
CP8	972	724	43 (4.42%)	205 (21.09%)	248 (23.51%)
TL	558	380	40 (7.71%)	195 (24.73%)	178 (31.90%)
TLP0	558	393	42 (7.53%)	232 (22.04%)	165 (29.57%)
TLP1	558	398	50 (8.96%)	209 (20.25%)	160 (28.67%)
TLP2	558	396	45 (8.06%)	236 (20.97%)	162 (29.30%)

Note: L, LP0, LP3, LP6: citrus leaf, citrus mesophyll protoplasts after 0, 3, and 6 days of culture; C, CP0, CP4, CP8: citrus callus, citrus callus protoplasts after 0, 4, and 8 days of culture; TL, TLP0, TLP1, TLP2: tobacco leaf, tobacco mesophyll protoplasts after 0, 1, and 2 days of culture.

**Table 2:** Citrus genomic DNA methylation modification sequence analysis results.

No.	Fragment Length (bp)	BLASTn Homolog Sequence Annotation	Similarity %	E Value
P8	111	Linoleate 13S-lipoxygenase 3-1, chloroplastic	65/65 (100%)	1 × 10^−29^
P9	124	CBL-interacting serine/threonine–protein kinase 14, regulatory pathway	123/123 (100%)	3 × 10^−64^
P14	90	Hypothetical protein	75/88 (85%)	2 × 10^−12^
P15	48	Xylem cysteine proteinase 1, chymopapain, papaya proteinase 4, caricain, papain, oryzain alpha chain	46/48 (95%)	3 × 10^−15^
P20	295	Putative uncharacterized protein Sb01g000365 (fragment)	293/293 (100%)	1 × 10^−165^
P23	92	E3 ubiquitin–protein ligase RNF12-B, E3 ubiquitin–protein ligase RLIM, putative RING-H2 finger protein ATL53	90/90 (100%)	9 × 10^−45^
P25	121	Putative uncharacterized protein Sb03g046360, NAD kinase 2, chloroplastic, probable NAD kinase 2, chloroplastic	119/121 (98%)	2 × 10^−58^
P28	97	Calcium-dependent protein kinase isoform 2, calcium-dependent protein kinase 33	37/37 (100%	4 × 10^−13^
P19	112	LRR receptor-like serine/threonine–protein kinase FLS2,	112/112 (100%)	9 × 10^−58^
P30	45	chain A, three-dimensional structure of an RNA polymerase II binding protein	44/44 (100%)	1 × 10^−17^
P32	294	Hydroxycinnamoyl CoA shikimate/quinate hydroxycinnamoyltransferase-like protein (fragment),	248/249 (99%)	1 × 10^−137^
P39	51	Myosin-J heavy chain	51/51 (100%)	8 × 10^−22^
P46	229	U micrococcal nuclease, protein parB, probable endonuclease LCL3, nuclease (SNase domain-containing protein) (precursor)	228/229 (99%)	1 × 10^−125^
P53	86	Leucine-rich repeat containing protein, putative, putative disease resistance protein RGA3	81/82 (98%)	1 × 10^−37^
P55	46	Glycoside hydrolase family 28 protein/polygalacturonase (pectinase) family protein	46/46 (100%)	7 × 10^−19^

**Table 3:** Sequence of adapters and primers of pre-amplification and alternative amplification used for MSAP analysis.

Adaptor and Primer (5′→3′)	Sequence (5′→3′)	Adaptor and Primer (5′→3′)	Sequence (5′→3′)
EcoR I adapter-5	CTCGTAGACTGCGTACC	H+M+TAT	ATCATGAGTCCTGCTCGGTAT
EcoRI adapter-3	AATTGGTACGCAGTCTAC	H+M+TCT	ATCATGAGTCCTGCTCGGTCT
Hpa II- Msp I adapter-5	GATCATGAGTCCTGCT	H+M+TGT	ATCATGAGTCCTGCTCGGTGT
Hpa II- Msp I adapter-3	CGAGCAGGACTCATGA	EA01	GACTGCGTACCAATTCATC
H+M+T	ATCATGAGTCCTGCTCGGT	EA02	GACTGCGTACCAATTCATG
EA00	GACTGCGTACCAATTCA	EA03	GACTGCGTACCAATTCACC
H+M+TAC	ATCATGAGTCCTGCTCGGTAC	EA04	GACTGCGTACCAATTCAGG
H+M+TAG	ATCATGAGTCCTGCTCGGTAG	EA05	GACTGCGTACCAATTCAAC
H+M+TTC	ATCATGAGTCCTGCTCGGTTC	EA06	GACTGCGTACCAATTCAAG
H+M+TTG	ATCATGAGTCCTGCTCGGTTG	EA07	GACTGCGTACCAATTCACT
H+M+TCC	ATCATGAGTCCTGCTCGGTCC	EA08	GACTGCGTACCAATTCAGT
H+M+TGG	ATCATGAGTCCTGCTCGGTGG	EA09	GACTGCGTACCAATTCAAT
H+M+TCA	ATCATGAGTCCTGCTCGGTCA	EA10	GACTGCGTACCAATTCATA
H+M+TGA	ATCATGAGTCCTGCTCGGTGA	EA11	GACTGCGTACCAATTCACA
H+M+TTA	ATCATGAGTCCTGCTCGGTTA	EA12	GACTGCGTACCAATTCAGA

## Data Availability

The data presented in this study can be found in the article.
